# From Pediatric to Adult HIV Care: What Works to Keep Adolescents Engaged—A Systematic Review of Transition Strategies

**DOI:** 10.3390/tropicalmed10100295

**Published:** 2025-10-16

**Authors:** Po-Pin Hung, Hsi-Hsien Wei

**Affiliations:** 1 Division of Infectious Diseases, Department of Internal Medicine, Taipei Tzu Chi Hospital, Buddhist Tzu Chi Medical Foundation, Taipei 23142, Taiwan; 2 Division of Infectious Diseases, Department of Pediatrics, Taipei Tzu Chi Hospital, Buddhist Tzu Chi Medical Foundation, Taipei 23142, Taiwan; 3 Institute of Emergency and Critical Care Medicine, National Yang Ming Chiao Tung University, Taipei 112304, Taiwan

**Keywords:** adolescent HIV, healthcare transition, retention in care, viral suppression, youth-friendly services, transition interventions, systematic review

## Abstract

Adolescents living with HIV face unique challenges when transitioning from pediatric to adult care—including stigma, disclosure concerns, and loss of support—that undermine continuity. We systematically searched PubMed, Embase, and Web of Science through August 2025 in accordance with PRISMA 2020. Two reviewers independently screened records and appraised risk of bias using Joanna Briggs Institute (JBI) tools by study design; appraisals informed interpretation but did not determine inclusion. Thirty-two studies met criteria; given heterogeneity, findings were narratively synthesized. Post-transition outcomes were suboptimal: retention often declined to ~70% by two years and viral suppression to roughly two-thirds, below global targets. Older transfer age and minimal gaps between pediatric discharge and adult enrollment were associated with better retention. Structured programs combining early preparation, coordinated handoffs, psychosocial/peer support, and youth-friendly adult services consistently improved engagement (often >90% 12-month retention), though improvements in viral suppression were less uniform. JBI appraisal indicated mostly moderate methodological quality with common risks from confounding and incomplete follow-up, tempering certainty. Purposeful, well-supported transition models are essential to sustain treatment success into adulthood; future work should evaluate scalable delivery and long-term outcomes across diverse settings.

## 1. Introduction

Globally, adolescents (ages 10–19) account for roughly 4% of all people living with HIV, and an estimated 11% of new HIV infections occur in this age group [1]. In 2023, approximately 1.55 million adolescents worldwide were living with HIV, but only about 65% were receiving antiretroviral therapy [2]. In the United States, youth 13–24 years old represent ~13% of new HIV diagnoses [3]. Due to the success of pediatric antiretroviral therapy, a growing cohort of perinatally and behaviorally infected adolescents are surviving into adulthood and require transition to adult HIV care [4]. Healthcare transition is defined as the purposeful, planned movement of adolescents from child-centered to adult-oriented care [5]. Effective transition is critical for maintaining continuity of care and long-term treatment outcomes, yet many adolescents and young adults experience difficulties during this process.

Transitioning from pediatric to adult HIV care presents numerous challenges. Adolescents often have longstanding bonds with their pediatric providers and multidisciplinary teams that have supported them throughout childhood [6]. They may fear leaving these trusted providers and worry about disclosing their HIV status anew to unfamiliar adult clinic staff [7]. Qualitative studies indicate that many adolescents do not know what to expect from adult care and dread having to re-tell their health history to new providers [8]. Additionally, pediatric and adult HIV clinics often differ in philosophy and setting—pediatric care tends to be family-centered and nurturing, whereas adult care expects more patient autonomy [5]. Communication gaps between pediatric and adult teams, fragmented care systems, and lack of clear transition protocols can further hinder the process [9].

Psychosocial factors add to the complexity. Many adolescents face intense HIV-related stigma and feel pressure to keep their diagnosis secret, which can isolate them and erode engagement in care. For instance, in a Southeast Asian cohort of 93 youth (median age ~20 years), two-thirds reported needing to hide their HIV status and about one-third had no confidant for personal problems [9]. Mental health issues (e.g., depression or anxiety) and the normal developmental challenges of adolescence (establishing independence, changing support networks) may negatively impact medication adherence and clinic attendance during the transition period. Indeed, studies have found that roughly half of youth report difficulties with adherence after moving to adult care, often citing psychosocial stressors or loss of support [4,6]. Without adequate preparation and support, many young people fall out of care shortly after transfer to adult clinics [10]. Such lapses during transition can lead to interruptions in treatment, loss of viral suppression, and even disease progression [10].

Recognizing these challenges, there is increasing emphasis on the need for structured transition processes for adolescents with HIV. International and national health agencies have developed formal guidelines to support adolescent transitions. For example, the American Academy of Pediatrics and partner organizations recommended in 2011 that all youth with special healthcare needs have a written transition plan, and an updated clinical report in 2018 reinforced these practices while highlighting the importance of individualized preparation [11]. In many low-resource settings explicit transition policies remain lacking and health systems struggle to implement standardized approaches. Overall, the literature underscores that planned, supported transition is critical for keeping adolescents engaged in care: when young people receive preparation and guidance, they are far more likely to remain in care throughout and after the transfer to adult services [12]. We systematically reviewed quantitative, qualitative, and mixed-methods studies to identify factors and transition strategies associated with improved engagement in care—particularly retention and viral suppression—among adolescents and young adults moving from pediatric to adult HIV services.

## 2. Methods

This systematic review was conducted in accordance with the PRISMA 2020 guidelines. The review was not prospectively registered.

We conducted a comprehensive literature search on the transition of adolescents from pediatric to adult HIV care, adhering to the Preferred Reporting Items for Systematic Reviews and Meta-Analyses (PRISMA 2020) guidelines. Multiple databases were systematically searched up to August 2025, including PubMed, Embase, and Web of Science. The search strategy used combinations of keywords and terms such as “adolescent HIV,” “transition to adult care,” “HIV care continuum,” “retention,” and “transition intervention.” We also hand-searched the reference lists of relevant papers and prior reviews to identify additional studies.

**Inclusion criteria:** We included studies (quantitative, qualitative, or mixed-methods) that involved adolescents or young adults living with HIV undergoing transition from pediatric to adult care, and that either reported post-transition outcomes (e.g., retention in care, viral suppression, mortality) or described a transition intervention or strategy. There were no geographic or regional restrictions, and all peer-reviewed articles (and select conference abstracts) published in English were considered.

**Exclusion criteria:** We excluded publications that did not address a pediatric-to-adult transition (for example, studies focusing only on pediatric or adult care with no transition), those that lacked any data on care engagement or health outcomes during/after transition, and commentary or opinion pieces without original data.

**Figure 1:**
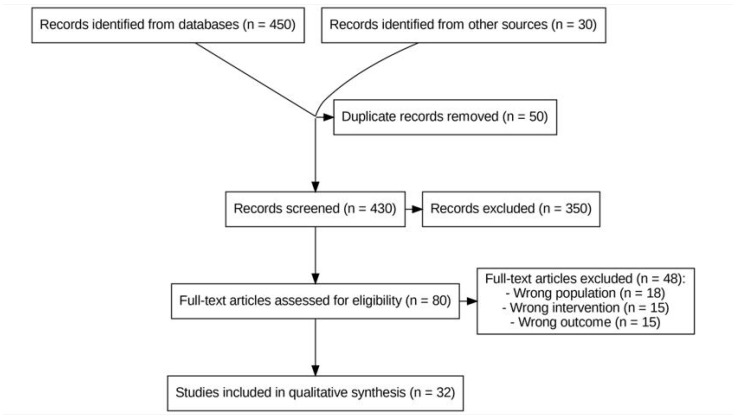
PRISMA 2020 flow diagram of study identification and selection (**n = 480** records identified [450 databases; 30 other sources]; **n = 50** duplicates removed; **n = 430** records screened; **n = 80** full-text articles assessed for eligibility; **n = 32** studies included).

After removing duplicates, two reviewers independently screened the titles and abstracts of the remaining 430 records, then evaluated 80 full-text articles for eligibility, yielding a final sample of 32 publications. The PRISMA 2020 flow diagram (Figure 1) summarizes the study selection process. In addition, two reviewers independently appraised the risk of bias of each included study using the Joanna Briggs Institute (JBI) Critical Appraisal Tool appropriate for its design (e.g., the JBI checklists for cohort studies, qualitative research, and randomized trials). Any discrepancies in appraisal were resolved by consensus. Importantly, no study was excluded based on the quality assessment results; instead, the critical appraisal findings were used to inform the narrative synthesis and interpretation of the evidence.

**Data extraction.** Two reviewers (P.-P.H. and H.-H.W.) independently extracted data from each included study using a pre-piloted, standardized form capturing study characteristics (design, setting, country, sample/age), transition model or intervention components, outcomes (retention in care, viral suppression, mortality), follow-up duration, and analytic notes (e.g., confounders, missing data). Discrepancies were resolved by discussion to produce a single harmonized dataset for synthesis.

Given the heterogeneity in study designs and outcome measures, we synthesized results narratively rather than performing a meta-analysis. We prioritized evidence from systematic reviews, multi-site cohorts, and randomized trials when available to support higher-level conclusions, while also incorporating illustrative findings from single-center and qualitative studies. The strength of evidence for various transition approaches is noted (e.g., observational vs. randomized trial data), and gaps in knowledge are highlighted to inform future research.

## 3. Results

### 3.1. Outcomes After Transition to Adult HIV Care

Numerous studies have documented suboptimal clinical outcomes for adolescents and young adults following the transition to adult HIV care. A recent systematic review and meta-analysis found that the pooled retention in care one year after transition was approximately 81%, which dropped to about 69% by two years post-transition [13]. In other words, nearly one-third of youth disengage from HIV care within two years of moving to adult services.

Individual cohort studies similarly report declines in retention and treatment adherence following transition. For example, an analysis of multi-site cohort data in the United States found that only ~55% of adolescents remained in care one year after transfer, and just 65% had achieved viral suppression at that time [14]. A retrospective cohort study in Atlanta noted that fewer than half of young adults were retained in care 2–3 years post-transition, with many experiencing lapses in medication adherence accompanied by detectable viral loads [7]. These findings underscore the vulnerability of adolescents during the transition period.

There is considerable variability in outcomes across different settings. Some clinics have reported relatively high short-term retention—for instance, one European center noted around 87–92% of adolescents remained in care one year post-transition [15]. In contrast, other programs have found much lower retention rates, especially over longer-term follow-up [15,16]. Viral suppression rates tend to mirror retention patterns: most reports show a decline in the proportion of youth with suppressed HIV RNA after transition to adult care. For context, UNAIDS has set the ambitious 95-95-95 targets (95% of people on therapy achieving viral suppression, among other goals) as a benchmark for ending the HIV epidemic [1]. Transitioning adolescents fall far below this benchmark—a pooled analysis estimated that only around two-thirds of transitioning adolescents maintain viral suppression after two years, well below the UNAIDS 95% viral suppression target for those on therapy [13,16]. Adolescents who fall out of care often experience rebound viremia and immune deterioration, highlighting that a successful transition is critical for long-term health outcomes.

Notably, certain factors have been associated with better post-transition outcomes. Older age at the time of transfer (for example, transitioning in the early 20s rather than the late teens) has been linked to higher retention in some studies, possibly reflecting greater maturity and readiness for adult care. Real-world data support this—in South Africa, a natural experiment compared adolescents who transitioned to adult care at age 12 (under an old policy) versus those who remained in pediatric care until they were older (under a new policy). Among those forced to transition at 12, only 49% were retained in care one year later, compared to 92% retention for peers who stayed in pediatric care until a later age (*p* < 0.001) [17]. This dramatic difference illustrates the benefit of postponing transition until the adolescent is developmentally ready.

Another factor correlated with improved outcomes is minimizing any gap between leaving pediatric care and enrolling in an adult clinic—a shorter (or zero) interruption in care during the transfer is associated with higher retention rates post-transition [18]. Conversely, youth with serious behavioral or psychosocial challenges—such as unstable housing, untreated mental health conditions, or substance use—are at higher risk of poor outcomes if those issues are not addressed before and during transition. For example, untreated depression or active substance use can undermine a young person’s ability to engage consistently in adult care.

Importantly, targeted interventions are being developed to improve these outcomes. One recent example is a cluster randomized trial in Kenya that evaluated a structured Adolescent Transition Package (ATP) to support youth during the move to adult care [19]. After one year, adolescents receiving the ATP intervention had significantly higher transition readiness scores than those in standard care (mean score 15.7 vs. 13.9, *p* = 0.024), including notably better HIV knowledge/literacy (mean domain score 4.0 vs. 3.0, *p* = 0.011). Although the trial did not observe short-term differences in viral suppression between groups, it demonstrated that structured preparation can substantially improve youths’ readiness to transition [19]. The authors concluded that “integrating [ATP] approaches could enhance long-term HIV care in youth as they age into adulthood,” underscoring how proactive transition interventions may impart skills that translate into better retention and virologic outcomes over time [19].

### 3.2. Psychosocial Factors

Beyond clinical metrics, a range of psychosocial factors influence transition outcomes. Adolescents often contend with stigma and secrecy around their HIV status, which can impede engagement. In one Asian cohort, two-thirds of youth felt a need to hide their HIV status [8]. Many also lose the extensive support systems that were in place during childhood; moving to adult care can coincide with reduced familial involvement and the challenge of navigating care more independently. In multiple studies, adolescents describe feeling unprepared and anxious about the unfamiliar adult clinic environment [8,20]. These factors contribute to disengagement and poor adherence: roughly half of adolescents in some cohorts report difficulty taking medications or keeping appointments after transition, commonly attributing this to psychosocial stressors, lack of support, or feeling overwhelmed [4,6]. Ensuring that adolescents have strong social support and mental health resources during the transition is therefore vital. If issues like depression, anxiety, or unstable housing are not addressed, they can derail even the best-planned transition.

Peer support and community engagement are frequently cited as ways to buffer these psychosocial stressors. Connecting adolescents with peers who have similar lived experiences can reduce feelings of isolation and build self-efficacy during transition. The World Health Organization and other experts emphasize that peer-driven, youth-friendly services—integrated with counseling and mental health support—can improve outcomes for adolescents with HIV [21]. In practice, many HIV programs facilitate peer support groups or mentor programs (“teen clubs,” camps, and support meetings) to help young people navigate the social and emotional aspects of living with HIV. Participants often report increased confidence, reduced stigma, and better understanding of their treatment. However, formal evaluations of peer support models show mixed effects on objective health outcomes. For example, in Namibia, adolescents who participated in clinic-based teen clubs had virtually identical 24-month retention (~90%) and viral suppression rates compared to those in standard care [22,23]. By contrast, more intensive peer-led models such as Zimbabwe’s community-based Zvandiri program have demonstrated clear benefits, showing significant reductions in virologic failure and improved retention [24].

### 3.3. Structural Barriers

Health system and structural factors can also facilitate or hinder successful transitions. Pediatric and adult HIV care differ in important ways: pediatric clinics tend to offer more nurturing, family-centered care with multidisciplinary support, whereas adult clinics often expect patients to navigate more autonomously [5]. This cultural shift can be jarring for young people. Communication gaps between pediatric and adult providers, fragmented medical record systems, and the absence of formal transition protocols can lead to adolescents “falling through the cracks” during the transfer [9]. High-income countries have increasingly established standard transition procedures (e.g., joint pediatric-adult case conferences, dedicated transition coordinators), but many low- and middle-income settings lack such protocols [10,11]. Several studies from sub-Saharan Africa note the absence of clear transition policies at the clinic or national level as a barrier to continuity [10,25].

Another critical barrier is access to sexual and reproductive health (SRH) services. Many adolescents living with HIV have needs related to contraception, pregnancy prevention, and STI screening. Integrating SRH services with HIV care provides convenient, one-stop access that is youth-friendly and confidential. However, implementation has been uneven. In Kenya, 11% of large clinics reported never providing condoms to adolescent patients, and 14% provided no contraceptive services [26]. Many adolescents still received primarily abstinence-based counseling, despite expressed needs for broader SRH support. Similar gaps in comprehensive SRH services have been observed across sub-Saharan Africa [27]. Without integrated services, adolescents may disengage from care entirely.

Legal and policy frameworks also shape transitions. Rigid age-of-consent laws for health services may impede adolescents’ autonomy and willingness to engage in care. Encouragingly, in sub-Saharan Africa, the proportion of countries allowing HIV testing for adolescents without parental consent increased from 37% in 2013 to 63% in 2021 [22]. Aligning national policies with adolescent needs—by lowering age-of-consent thresholds, expanding confidentiality protections, and officially recognizing the unique needs of transitioning youth—is a key structural strategy.

### 3.4. Programmatic Strategies

In response to these challenges, a variety of transition models and interventions have been developed and tested. Best practices identified for facilitating successful transition include beginning the process early, providing comprehensive preparation and education, fostering clear communication between pediatric and adult providers, and ensuring ongoing support during and after the transfer [12]. Many pediatric HIV programs now implement individualized transition plans and formal readiness assessments to gauge each adolescent’s preparedness for adult care [11,21]. For example, providers may use checklists or readiness questionnaires to determine if a youth has the knowledge and skills needed to navigate adult clinics (such as understanding their medications and knowing how to schedule appointments). Early introduction of the adult care team—for instance, arranging for an adult HIV provider or nurse to meet the adolescent while they are still in pediatric care—can help build trust and familiarity before the transfer [11,21]. Multidisciplinary involvement is another key element: social workers, counselors, and peer navigators can address the holistic needs of adolescents during transition, from mental health support to assistance with insurance or transportation.

Several structured transition programs have reported encouraging results. One notable example is the Stepping Up (STEP) Program at the University of Maryland, which integrates an adult HIV clinician and a transition navigator directly into the pediatric clinic. Youth enrolled in STEP receive gradual orientation to the adult clinic and a formal handoff involving both pediatric and adult care teams. Outcomes from STEP have been excellent: about 95% of youth in the program were still engaged in adult care 12 months after transfer, compared to only ~50% in a comparable pre-STEP cohort [28]. Similarly, the Adolescent and Young Adult “Bridge” Clinic at Vanderbilt achieved 95.5% one-year retention, with viral suppression rates improving from 66.7% at entry to 81.0% at last follow-up [29].

In Thailand, the Happy Teen Program developed a holistic transition process that included group sessions with adult providers prior to transfer. This model yielded 87% one-year retention and 77% viral suppression [20]. In Italy, a multicenter pilot protocol that combined phased education, joint pediatric–adult consultations, and structured follow-up achieved 92% retention and 92% viral suppression at 18 months [21]. These diverse examples demonstrate that with intentional design, successful transition is possible even across varied contexts.

Common elements of effective transition programs include:

- Early preparation and readiness assessments.
- Structured handoffs with direct communication between pediatric and adult teams.
- Ongoing psychosocial and mental health support.
- Youth-friendly adult clinic environments.
- Use of digital tools such as reminder apps and social media outreach.

**Figure 2:**
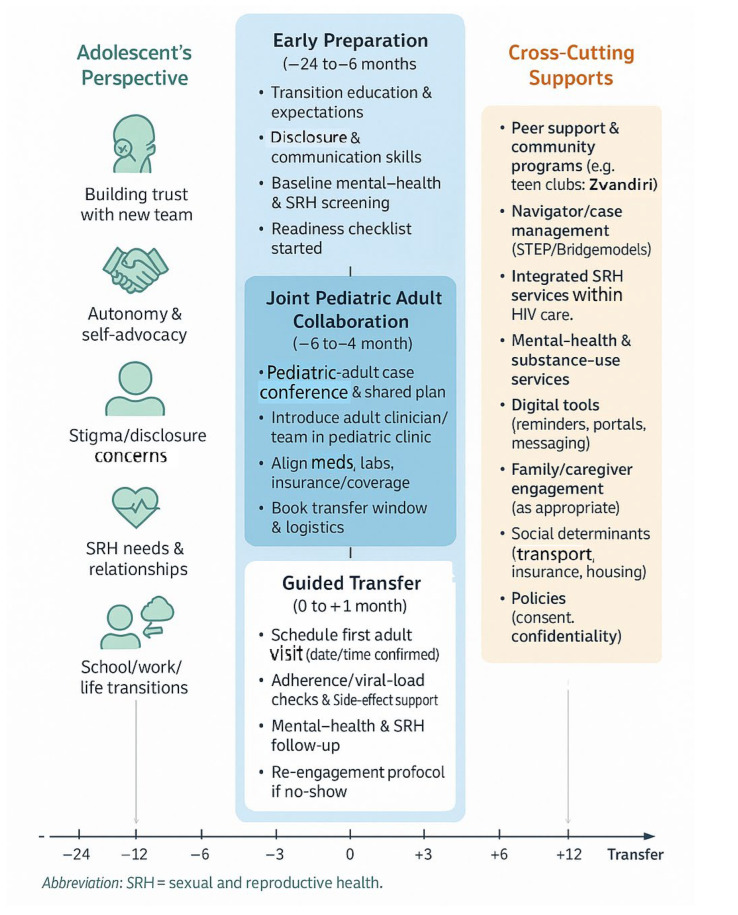
Staged Transition Framework for Adolescents Moving from Pediatric to Adult HIV Care. This framework illustrates key phases from early preparation to guided transfer and post-transition support, alongside cross-cutting elements like peer support and integrated sexual and reproductive health (SRH) services.

Together, these elements inform a five-stage, youth-centered transition framework (Figure 2), with exemplar programs summarized in Table 1.

### 3.5. Risk of Bias Assessment

Using the Joanna Briggs Institute (JBI) Critical Appraisal Tools, we assessed the methodological quality and risk of bias of all 32 included studies. Overall, seven studies were rated as low risk of bias, twenty as moderate, and five as high risk. Common limitations included small sample sizes, inadequate control for confounding, and short follow-up periods. Detailed JBI appraisal results are available in the Supplementary Materials.

## 4. Discussion

The evidence reviewed shows that the transition from pediatric to adult HIV care is a pivotal juncture that can greatly influence long-term outcomes for adolescents living with HIV. Without structured support, many youths experience interruptions in care and treatment during this period, leading to worsened clinical outcomes. Transition is not a one-time event but rather a process unfolding over months to years, requiring coordination, communication, and sustained support. Adolescents are not simply “graduating” to adult care; they are entering an unfamiliar system at a developmental stage already fraught with change. Accordingly, effective transition programs treat it as a gradual process, beginning well before transfer and continuing after with follow-up and re-engagement efforts.

Targeted interventions can dramatically improve retention in care for transitioning youth. Multidisciplinary, youth-focused programs—whether integrated pediatric–adult clinic models, dedicated transition clinics, or intensive case management approaches—consistently demonstrate higher retention (often >90% at 1 year) compared to historical norms under usual care (typically 50–70%) [28]. Importantly, these interventions often require significant resources: extra staff time for transition coordination, smaller patient caseloads to allow individualized attention, and sometimes technology or peer support components. This raises questions about scalability, especially in low-resource settings. Implementation science must address whether task shifting (using lay counselors or peer mentors) or mobile health tools can achieve comparable results [39].

Another noteworthy point is that improved retention in care does not automatically equate to improved viral suppression. Some interventions, such as the ACE youth-focused track in Baltimore, achieved better retention but saw persistent gaps in viral suppression [32]. This suggests that keeping young people in care, while necessary, is not sufficient. Youth-friendly services should integrate adherence support, mental healthcare, and substance use services to address barriers that continue to impede suppression after transition. Essentially, the quality and youth-appropriateness of adult HIV care will determine whether retained youth actually realize health benefits. Adult HIV providers may need additional training to adopt adolescent-friendly practices, such as providing more intensive counseling, using communication styles that resonate with younger patients, and involving family or caregivers (with consent).

The current evidence base also has limitations. Many included studies were observational, with small sample sizes and short follow-up durations. In line with these issues, our JBI-based critical appraisal found that most of the included studies were of only moderate quality, with several rated at high risk of bias. This overall risk of bias in the evidence base limits our confidence in the findings—any reported benefits of transition interventions should be interpreted with caution given the methodological weaknesses of the underlying studies. Few randomized trials have rigorously tested transition interventions, and those often focus on readiness rather than long-term outcomes [31]. Reporting bias may be present, as successful programs are more likely to be published. A 2016 Cochrane review concluded that there was a lack of high-quality evidence to inform best practices for healthcare transition in chronic illnesses, including HIV [31]. While the field has progressed since then—with systematic reviews on specific aspects such as retention [13], digital health interventions [39], and youth care models [37]—gaps remain, especially in low-resource settings. Data on long-term (>5 years) outcomes after transition are scarce, and studies often focus on perinatally infected populations, with limited data on behaviorally infected youth. More qualitative research is needed to capture adolescents’ voices, emphasizing what they find most helpful or harmful during transition [26].

A growing body of research examines self-management and empowerment interventions. Self-management support is intuitively beneficial, helping youth build skills in adherence, appointment-keeping, and self-advocacy. However, evidence for effectiveness is limited. A systematic review of self-management interventions for adolescents with HIV concluded that such programs have uncertain effects on outcomes [36]. This underscores that educational and skills-building efforts alone may not be enough; without addressing the broader psychosocial and systemic context, their impact may be modest. Programs must pair empowerment with peer support, mental health services, and enabling clinic environments.

## 5. Conclusions

The transition from pediatric to adult HIV care is a sensitive period carrying both challenges and opportunities. Without deliberate planning and support, many adolescents experience disruptions that compromise hard-won treatment gains. However, structured strategies and dedicated programs can mitigate these risks. When healthcare systems implement multidisciplinary, youth-focused interventions characterized by early preparation, coordinated handoffs, psychosocial support, and individualized planning, improved retention and suppression rates are achievable.

Scaling up evidence-informed transition models is crucial, particularly in resource-limited settings where most adolescents with HIV live. This includes training adult HIV providers in adolescent-friendly practices, embedding formal transition protocols in health systems, and continually evaluating outcomes to refine approaches. Future research priorities include long-term outcome studies, rigorous comparative trials of transition interventions, and evaluation of emerging approaches such as digital health tools and integrated sexual/reproductive health services.

Ultimately, smoothing the path from pediatric to adult care will help ensure that treatment successes achieved in childhood are not lost in adolescence and young adulthood. A successful transition is not an endpoint but a foundation for lifelong engagement in care.

## Figures and Tables

**Table 1:** Summary of Included Studies by Factor/Intervention Type and Key Findings.

Citation	Key Findings	Focus	Study (Author, Year, Country/Setting; Design)
			**Individual Factors**
[14]	55% retained; 65% suppressed after 1 year.	Outcomes after transition.	Tanner et al., 2018 (USA, multi-site; prospective cohort)
[7]	<50% retained; many lost suppression.	Retention 2–3 years post-transition.	Hussen et al., 2017 (USA, Atlanta; retrospective cohort)
[4]	Over half had unsuppressed viremia during transition.	Perinatally infected youth.	Tassiopoulos et al., 2020 (USA, national; prospective cohort)
[15]	84% retained long-term.	Real-life transition clinic.	Righetti et al., 2015 (Italy, Genoa; cohort)
[30]	~88% retained; strong suppression.	Specialized young adult clinic.	Rungmaitree et al., 2022 (Thailand, Bangkok; cohort)
			**Psychosocial Factors**
[9]	2/3 concealed status; 1/3 lacked confidants.	Stigma and disclosure.	Sohn et al., 2020 (SE Asia; cohort)
[25]	Poor preparation linked to poor retention.	Disclosure timing and support.	Njuguna et al., 2019 (Kenya, multicenter)
[8]	Youth valued trust, support, and gradual preparation.	Qualitative review.	Varty et al., 2020 (Global metasynthesis)
			**Structural Barriers**
[17]	Transition at 12 yrs → 49% retained vs. 92% if older.	Policy-based early transfer.	Zanoni et al., 2020 (South Africa; natural experiment)
[18]	Delays worsened retention; seamless linkage important.	Care gaps.	Ryscavage et al., 2016 (USA, Baltimore; cohort)
[12]	Wide variation; lack of readiness assessments.	Transition processes.	Tanner et al., 2015 (USA; survey of 12 clinics)
[10]	Transitions often unplanned; poor retention.	Regional practices.	Dahourou et al., 2017 (Sub-Saharan Africa; review)
[16]	Wide variation; suppression ~62%.	Retention outcomes.	Ritchwood et al., 2020 (Global systematic review)
[31]	Limited HIV-specific evidence.	Chronic illness transitions.	Campbell et al., 2016 (Global Cochrane review)
			**Programmatic Interventions**
[28]	95% retention with integrated adult provider and navigator vs. 50% in pre-program cohort.	Integrated pediatric–adult care transition program (STEP).	Ryscavage et al., 2022 (USA, Maryland; STEP program)
[32]	Youth track halved LTFU; 2-yr retention 49% vs. 26%.	Youth-dedicated clinic track (ACE).	Griffith et al., 2019 (USA, Baltimore; ACE clinic)
[29]	95.5% retention; suppression improved from 66.7% to 81%.	Structured transition bridge clinic (Bridge).	Chew et al., 2024 (USA, Vanderbilt; Bridge clinic)
[20]	87% retention; 77% suppression at 12 months.	Happy Teen adolescent support program.	Lolekha et al., 2017 (Thailand; Happy Teen program)
[21]	92% retention and suppression at 18 months.	Pilot multidisciplinary transition clinic.	Continisio et al., 2018 (Italy; multicenter pilot)
[33]	Peer support feasible and acceptable; no outcomes yet.	Tikambisane peer support group.	Stangl et al., 2021 (Zambia; Tikambisane)
[24]	Peer counselors cut virologic failure/death by 42%.	Zvandiri peer counseling intervention.	Mavhu et al., 2020 (Zimbabwe; Zvandiri trial)
[34]	Improved ART uptake and clinic attendance.	Project ACCEPT peer-support intervention.	Hosek et al., 2018 (USA; Project ACCEPT RCT)
[22]	~90% retention, no significant difference vs. standard care.	Clinic-based teen clubs.	Munyayi et al., 2020 (Namibia; teen clubs)
[23]	Teen clubs did not improve suppression.	Clinic-based teen clubs.	Munyayi et al., 2020 (Namibia; comparative)
[35]	Navigation and case management improved engagement.	Patient navigation and case management.	Momplaisir et al., 2023 (USA, Philadelphia)
[36]	Self-management interventions show uncertain effect.	Systematic review of self-management interventions.	Crowley & Rohwer, 2021 (Global; review)
[19]	Improved readiness; no virologic effect short-term.	Adolescent Transition Package (ATP) trial.	Njuguna et al., 2022 (Kenya; ATP trial)
[37]	Youth-tailored interventions improve retention.	Narrative review of youth-tailored interventions.	Mulawa et al., 2023 (Global; narrative review)
[38]	Case management and peer support improved retention.	Multisite transition program evaluation.	Griffith et al., 2019 (USA; multisite pre/post)

Abbreviations: ART, antiretroviral therapy; LTFU, lost to follow-up; RCT, randomized controlled trial; ATP, Adolescent Transition Package; STEP, Stepping Up transition program; ACE, youth-dedicated “ACE” clinic (program name); mHealth, mobile health; SE Asia, Southeast Asia.

## Data Availability

No new data were created or analyzed in this study. All data are contained within the article and its cited references. Screening/extraction forms and any intermediate datasets are available from the corresponding author on reasonable request.

## Supplementary Materials

The following supporting information can be downloaded at: https://www.mdpi.com/article/10.3390/tropicalmed10100295/s1, Supplementary Table S1. JBI Critical Appraisal—Summary by Study.

## References

[1] UNAIDS (2024). Global HIV & AIDS Statistics—Fact Sheet. https://www.unaids.org/en/resources/fact-sheet.

[2] UNICEF (2023). Adolescent HIV prevention—Global HIV Statistics (2023). https://data.unicef.org/topic/hivaids/adolescents-young-people/.

[3] Centers for Disease Control and Prevention (CDC) (2023). HIV and Youth: HIV in the United States by Age. https://www.cdc.gov/hiv-data/nhss/estimated-hiv-incidence-and-prevalence.html.

[4] Tassiopoulos K., Huo Y., Patel K., Kacanek D., Allison S., Siminski S., Nichols S.L., Mellins C.A. (2020). Healthcare transition outcomes among young adults with perinatally acquired HIV in the United States. Clin. Infect. Dis..

[5] Blum R.W., Garell D., Hodgman C.H., Jorissen T.W., Okinow N.A., Orr D.P., Slap G.B. (1993). Transition from child-centered to adult healthcare systems for adolescents with chronic conditions. J. Adolesc. Health.

[6] Cervia J.S. (2013). Easing the transition of HIV-infected adolescents to adult care. AIDS Patient Care STDs.

[7] Hussen S.A., Chakraborty R., Knezevic A., Camacho-Gonzalez A., Huang E., Stephenson R., del Rio C. (2017). Transitioning young adults from pediatric to adult care and the HIV care continuum in Atlanta, Georgia, USA: A retrospective cohort study. J. Int. AIDS Soc..

[8] Varty M., Speller-Brown B., Phillips L., Kelly K.P. (2020). Youths’ experiences of transition from pediatric to adult care: An updated qualitative metasynthesis. J. Pediatr. Nurs..

[9] Sohn A.H., Chokephaibulkit K., Lumbiganon P., Hansudewechakul R., Gani Y.M., Van Nguyen L., Mohamed T.J., Teeraananchai S., Sethaputra C., Singtoroj T. (2020). Peritransition outcomes of Southeast Asian adolescents and young adults with HIV transferring from pediatric to adult care. J. Adolesc. Health.

[10] Dahourou D.L., Gautier-Lafaye C., Teasdale C.A., Renner L., Yotebieng M., Desmonde S., Ayaya S., Davies M., Leroy V. (2017). Transition from paediatric to adult care of adolescents living with HIV in sub-Saharan Africa: Challenges, youth-friendly models, and outcomes. J. Int. AIDS Soc..

[11] White P.H., Cooley W.C., Transitions Clinical Report Authoring Group (2018). Supporting the health care transition from adolescence to adulthood in the medical home. Pediatrics.

[12] Tanner A.E., Philbin M.M., Duval A., Ellen J., Kapogiannis B., Fortenberry J.D. (2016). Transitioning HIV-Positive Adolescents to Adult Care: Lessons Learned From Twelve Adolescent Medicine Clinics. J. Pediatr. Nurs..

[13] Shimbre M.S., Belete A.G., Guyo T.G., Ma W. (2025). Retention in care after transition to adult care for adolescents and young adults with HIV: A systematic review and meta-analysis. Int. J. Public. Health.

[14] Tanner A.E., Philbin M.M., Chambers B.D., Ma A., Hussen S., Ware S., Lee S., Fortenberry J.D. (2018). Healthcare transition for youth living with HIV: Outcomes from a prospective multi-site study. J. Adolesc. Health.

[15] Righetti A., Prinapori R., Nulvesu L., Fornoni L., Viscoli C., Di Biagio A. (2015). Transitioning HIV-infected children and adolescents into adult care: An Italian real-life experience. J. Assoc. Nurses AIDS Care.

[16] Ritchwood T.D., Malo V., Jones C., Metzger I.W., Atujuna M., Marcus R., Conserve D.F., Handler L., Bekker L.-G. (2020). Healthcare retention and clinical outcomes among adolescents living with HIV after transition from pediatric to adult care: A systematic review. BMC Public Health.

[17] Zanoni B.C., Archary M., Sibaya T., Musinguzi N., Haberer J.E. (2020). Transition from pediatric to adult care for adolescents living with HIV in South Africa: A natural experiment and survival analysis. PLoS ONE.

[18] Ryscavage P., Macharia T., Patel D., Palmeiro R., Tepper V. (2016). Linkage to and retention in care following healthcare transition from pediatric to adult HIV care. AIDS Care.

[19] Njuguna I.N., Beima-Sofie K., Mburu C.W., Mugo C., Itindi J., Onyango A., Neary J., Richardson B.A., Oyiengo L., Wamalwa D. (2022). Transition to independent care for youth living with HIV: A cluster randomised clinical trial. Lancet HIV.

[20] Lolekha R., Boonyasidhi V., Na-Nakorn Y., Manaboriboon B., Vandepitte W.P., Martin M., Tarugsa J., Nuchanard W., Leowsrisook P., Lapphra K. (2017). The Happy Teen Programme: A holistic outpatient clinic-based approach to prepare HIV-infected youth for transition to adult care. J. Int. AIDS Soc..

[21] Continisio G.I., Lo Vecchio A., Basile F.W., Russo C., Cotugno M.R., Palmiero G., Storace C., Mango C., Guarino A., Bruzzese E. (2020). The Transition of Care From Pediatric to Adult Healthcare Services of Vertically HIV-Infected Adolescents: A Pilot Study. Front. Pediatr..

[22] Munyayi F.K., van Wyk B. (2020). The effects of teen clubs on retention in HIV care among adolescents in Windhoek, Namibia. S Afr. J. HIV Med..

[23] Munyayi F.K., van Wyk B. (2020). The comparison of teen clubs vs. standard care on treatment outcomes for adolescents on antiretroviral therapy in Windhoek, Namibia. AIDS Res. Treat..

[24] Mavhu W., Willis N., Mufuka J., Bernays S., Tshuma M., Mangenah C., Maheswaran H., Mangezi W., Apollo T., Araya R. (2020). Effect of a differentiated service delivery model on virological failure in adolescents with HIV in Zimbabwe (Zvandiri): A cluster-randomised controlled trial. Lancet Glob. Health.

[25] Njuguna I.N., Beima-Sofie K., Mburu C.W., Mugo C., Itindi J., Onyango A., Wamalwa D., Black A.D., Neary J., Slyker J. (2019). Managing the transition from paediatric to adult care for HIV, Kenya. Bull. World Health Organ..

[26] Lawrence B., Moraa E., Wilson K.S., Mutisya M., Neary J., Kinuthia J., Itindi J., Nyaboe E., Muhenje O., Chen T.-H. (2021). “They just tell me to abstain”: Variable access to and uptake of sexual and reproductive health services among adolescents living with HIV in Kenya. Front. Reprod. Health.

[27] Chem E.D., Ferry A., Seeley J., Weiss H.A., Simms V. (2022). Health-related needs reported by adolescents living with HIV and receiving antiretroviral therapy in sub-Saharan Africa: A systematic literature review. J. Int. AIDS Soc..

[28] Ryscavage P., Herbert L., Roberts B., Cain J., Lovelace S., Houck D., Tepper V. (2022). Stepping Up: Retention in HIV care within an integrated health care transition program. AIDS Care.

[29] Chew H.K., Bonnet K., Schlundt D.G., Hill N.S., Desai N. (2024). Mixed-Methods Evaluation of a Youth-Friendly Clinic for Young People Living With HIV Transitioning From Pediatric Care. Trop. Med. Infect. Dis..

[30] Rungmaitree S., Thamniamdee N., Sachdev S., Phongsamart W., Lapphra K., Wittawatmongkol O., Maleesatharn A., Khumcha B., Hoffman R.M., Chokephaibulkit K. (2022). Outcomes of transition from pediatrics to adult care among adolescents and young adults with HIV at a tertiary care center in Bangkok. J. Int. Assoc. Provid. AIDS Care.

[31] Campbell F., Biggs K., Aldiss S.K., O’Neill P.M., Clowes M., McDonagh J., While A., Gibson F. (2016). Transition of care for adolescents from pediatric services to adult health services. Cochrane Database Syst. Rev..

[32] Griffith D., Snyder J., Dell S., Nolan K., Keruly J., Agwu A. (2019). Impact of a Youth-Focused Care Model on Retention and Virologic Suppression Among Young Adults With HIV in an Adult Clinic. J. Acquir. Immune Defic. Syndr..

[33] Stangl A.L., Mwale M., Sebany M., Mackworth-Young C.R., Chiiya C., Chonta M., Clay S., Sievwright K., Bond V. (2021). Feasibility, acceptability and preliminary efficacy of Tikambisane (“Let’s Talk to Each Other”): A pilot support group intervention for adolescent girls living with HIV in Zambia. J. Int. Assoc. Provid. AIDS Care.

[34] Hosek S.G., Harper G.W., Lemos D., Burke-Miller J., Lee S., Friedman L., Martinez J. (2018). Project ACCEPT: Evaluation of a group-based intervention to improve engagement in care for youth newly diagnosed with HIV. AIDS Behav..

[35] Momplaisir F., McGlonn K., Grabill M., Moahi K., Nkwihoreze H., Knowles K., Laguerre R., Dowshen N., Hussen S.A., Tanner A.E. (2023). Strategies to improve outcomes of youth experiencing healthcare transition from pediatric to adult HIV care in a large U.S. city. Arch. Public. Health.

[36] Crowley T., Rohwer A. (2021). Self-management interventions for adolescents living with HIV: A systematic review. BMC Infect. Dis..

[37] Mulawa M.I., Knippler E.T., Al-Mujtaba M., Wilkinson T.H., Ravi V.K., Ledbetter L.S. (2023). Interventions to improve adolescent HIV care outcomes. Curr. HIV/AIDS Rep..

[38] Griffith D., Bhalakia A., Buck A., Agwu A. (2019). Improving retention in adult HIV care for youth: A retrospective multisite analysis of the impact of a transition-focused clinic and care navigation support. Pediatr. Infect. Dis. J..

[39] Goldstein M., Archary M., Adong J., Haberer J.E., Kuhns L.M., Kurth A., Ronen K., Lightfoot M., Inwani I., John-Stewart G. (2023). Systematic review of mHealth interventions for adolescent and young adult HIV prevention and the HIV continuum of care in low- to middle-income countries. AIDS Behav..

