# Supplementary material for: From Pediatric to Adult HIV Care: What Works to Keep Adolescents Engaged—A Systematic Review of Transition Strategies
Source: Trop Med Infect Dis. 2025 Oct 16;10(10):295. doi: 10.3390/tropicalmed10100295 (PMC12567669; doi:10.3390/tropicalmed10100295)
Supplement: Supplementary file 1 [file tropicalmed-10-00295-s001.zip › tropicalmed-3861794-Supplementary.pdf]

Supplementary Table S1. JBI Critical Appraisal — Summary by Study

Two reviewers appraised studies independently using JBI Critical Appraisal Tools appropriate to design; disagreements were resolved by consensus.

Overall Appraisal rule: High if any domain High; Low only if all domains Low; otherwise Moderate. Ref # corresponds to the main-text reference list.

Study (Author, Year)	Country/Setting	Study Design	JBI Tool Used	Overall Appraisal	Ref #	Key Limitations / Notes
**Tanner et al., 2018**	USA (multisite)	Prospective cohort	JBI Cohort	Moderate	[14]	Unadjusted confounding; incomplete follow-up; limited comparators.
**Hussen et al., 2017**	USA (Atlanta)	Retrospective cohort	JBI Cohort	High	[7]	Unadjusted confounding; incomplete follow-up; limited comparators.
**Tassiopoulos et al., 2020**	USA (national)	Prospective cohort	JBI Cohort	Low	[4]	Strengths: clear criteria, valid measurement, appropriate analysis, minimal attrition/confounding.
**Righetti et al., 2015**	Italy (Genoa)	Cohort	JBI Cohort	Low	[15]	Strengths: clear criteria, valid measurement, appropriate analysis, minimal attrition/confounding.
**Rungmaitree et al., 2022**	Thailand (Bangkok)	Cohort	JBI Cohort	Low	[30]	Strengths: clear criteria, valid measurement, appropriate analysis, minimal attrition/confounding.
**Sohn et al., 2020**	Southeast Asia (multi-country)	Cohort	JBI Cohort	Moderate	[9]	Unadjusted confounding; incomplete follow-up; limited comparators.
**Njuguna et al., 2019**	Kenya (multicenter)	Cross-sectional (analytical) (program report)	JBI Analytical Cross-sectional	High	[25]	Single timepoint; susceptibility to confounding; self-report measures.
**Varty et al., 2020**	Global	Systematic review of qualitative	JBI Systematic Review	Moderate	[8]	Heterogeneous methods; variable risk-of-bias reporting;

		studies	(qualitative)			registration/protocol not reported.
**Zanoni et al., 2020**	South Africa	Cohort (natural experiment)	JBI Cohort	Low	[17]	Strengths: clear criteria, valid measurement, appropriate analysis, minimal attrition/confounding.
**Ryscavage et al., 2016**	USA (Baltimore)	Cohort	JBI Cohort	Moderate	[18]	Unadjusted confounding; incomplete follow-up; limited comparators.
**Tanner et al., 2015**	USA (12 clinics)	Cross-sectional (analytical) (survey)	JBI Analytical Cross-sectional	Moderate	[12]	Single timepoint; susceptibility to confounding; self-report measures.
**Dahourou et al., 2017**	Sub-Saharan Africa	Systematic review	JBI Systematic Review	Moderate	[10]	Heterogeneous methods; variable risk-of-bias reporting; registration/protocol not reported.
**Ritchwood et al., 2020**	Global	Systematic review	JBI Systematic Review	Moderate	[16]	Heterogeneous methods; variable risk-of-bias reporting; registration/protocol not reported.
**Campbell et al., 2016**	Global	Systematic review (Cochrane)	JBI Systematic Review	Moderate	[31]	Heterogeneous methods; variable risk-of-bias reporting; registration/protocol not reported.
**Ryscavage et al., 2022**	USA (Maryland)	Pre–post (quasi-experimental) program evaluation (STEP)	JBI Quasi-experimental	Moderate	[28]	No concurrent control; potential baseline differences; short follow-up.
**Griffith et al., 2019**	USA (Baltimore)	Pre–post (quasi-experimental) youth-dedicated clinic (ACE)	JBI Quasi-experimental	Moderate	[32]	No concurrent control; potential baseline differences; short follow-up.
**Chew et al., 2024**	USA (Vanderbilt)	Mixed-methods evaluation	JBI Cohort + JBI Qualitative	Low	[29]	Strengths: clear criteria, valid measurement, appropriate analysis, minimal attrition/confounding.
**Lolekha et al., 2017**	Thailand	Pre–post (quasi-experimental) program	JBI Quasi-experimental	Moderate	[20]	No concurrent control; potential baseline differences; short

		evaluation (Happy Teen)				follow-up.
**Continisio et al., 2018**	Italy (multicenter)	Pilot program (pre–post, quasi-experimental)	JBI Quasi-experimental	High	[21]	No concurrent control; potential baseline differences; short follow-up.
**Stangl et al., 2021**	Zambia	Pre–post (quasi-experimental) feasibility pilot	JBI Quasi-experimental	High	[33]	No concurrent control; potential baseline differences; short follow-up.
**Mavhu et al., 2020**	Zimbabwe	Cluster RCT (Zvandiri)	JBI RCT (cluster design)	Low	[24]	Strengths: clear criteria, valid measurement, appropriate analysis, minimal attrition/confounding.
**Hosek et al., 2018**	USA	Group-based intervention (pre–post, quasi-experimental)	JBI Quasi-experimental	Moderate	[34]	No concurrent control; potential baseline differences; short follow-up.
**Munyayi et al., 2020**	Namibia (Windhoek)	Observational cohort (teen clubs)	JBI Cohort	Moderate	[22]	Unadjusted confounding; incomplete follow-up; limited comparators.
**Munyayi et al., 2020**	Namibia (Windhoek)	Comparative observational (teen clubs vs standard care)	JBI Cohort	High	[23]	Key concerns: confounding not controlled; incomplete follow-up; limited comparators.
**Momplaisir et al., 2023**	USA (Philadelphia)	Observational intervention (navigation/case management)	JBI Cohort	Moderate	[35]	Some concerns (confounding/attrition or measurement); analysis generally appropriate.
**Crowley & Rohwer, 2021**	Global	Systematic review	JBI Systematic Review	Moderate	[36]	Heterogeneous methods; variable risk-of-bias reporting; registration/protocol not reported.
**Njuguna et al., 2022**	Kenya	Cluster randomized trial (ATP)	JBI RCT (cluster design)	Low	[19]	Strengths: clear criteria, valid measurement, appropriate analysis, minimal attrition/confounding.
**Mulawa et al., 2023**	Global	Narrative review	JBI Systematic Review	Moderate	[37]	Heterogeneous methods; variable risk-of-bias reporting;

			(narrative)			registration/protocol not reported.
**Griffith et al., 2019**	USA (multisite)	Retrospective multisite pre–post (quasi-experimental)	JBI Quasi-experimental	Moderate	[38]	No concurrent control; potential baseline differences; short follow-up.
**Shimbre et al., 2025**	Global	Systematic review & meta-analysis	JBI Systematic Review	Moderate	[13]	Heterogeneous methods; variable risk-of-bias reporting; registration/protocol not reported.
**Lawrence et al., 2021**	Kenya	Cross-sectional (analytical) (SRH services)	JBI Analytical Cross-sectional	Moderate	[26]	Single timepoint; susceptibility to confounding; self-report measures.
**Chem et al., 2022**	Sub-Saharan Africa	Systematic review	JBI Systematic Review	Moderate	[27]	Heterogeneous methods; variable risk-of-bias reporting; registration/protocol not reported.

Abbreviations: JBI, Joanna Briggs Institute; RCT, randomized controlled trial; SRH, sexual and reproductive health; SR, systematic review.

Summary of overall ratings: Low = 7, Moderate = 20, High = 5 (matches Results §3.5).

Note: For mixed-methods (e.g., Chew 2024), Overall reflects the more conservative rating when component appraisals differed.
